# Biophysical impacts of northern vegetation changes on seasonal warming patterns

**DOI:** 10.1038/s41467-022-31671-z

**Published:** 2022-07-07

**Authors:** Xu Lian, Sujong Jeong, Chang-Eui Park, Hao Xu, Laurent Z. X. Li, Tao Wang, Pierre Gentine, Josep Peñuelas, Shilong Piao

**Affiliations:** 1 Sino-French Institute for Earth System Science, College of Urban and Environmental Sciences, Peking University, Beijing, China; 2 Department of Earth and Environmental Engineering, Columbia University, New York, NY USA; 3 Department of Environmental Planning, Graduate School of Environmental Studies, Seoul National University, Seoul, Republic of Korea; 4 Environmental Planning Institute, Seoul National University, Seoul, Republic of Korea; 5 Center for Sustainable Environment Research, Korea Institute of Science and Technology, Seoul, Republic of Korea; 6 Laboratoire de Météorologie Dynamique, CNRS, Sorbonne Université, Ecole Normale Supérieure, Ecole Polytechnique, Paris, France; 7 State Key Laboratory of Tibetan Plateau Earth System, Resources and Environment (TPESRE), Institute of Tibetan Plateau Research, Chinese Academy of Sciences, Beijing, China; 8 Earth Institute, Columbia University, New York, NY USA; 9 CREAF, Cerdanyola del Valles, Barcelona, Catalonia Spain; 10 CSIC, Global Ecology Unit CREAF-CSIC-UAB, Bellaterra, Barcelona, Catalonia Spain

**Keywords:** Environmental sciences, Ecology, Climate sciences

## Abstract

The seasonal greening of Northern Hemisphere (NH) ecosystems, due to extended growing periods and enhanced photosynthetic activity, could modify near-surface warming by perturbing land-atmosphere energy exchanges, yet this biophysical control on warming seasonality is underexplored. By performing experiments with a coupled land-atmosphere model, here we show that summer greening effectively dampens NH warming by −0.15 ± 0.03 °C for 1982–2014 due to enhanced evapotranspiration. However, greening generates weak temperature changes in spring (+0.02 ± 0.06 °C) and autumn (−0.05 ± 0.05 °C), because the evaporative cooling is counterbalanced by radiative warming from albedo and water vapor feedbacks. The dwindling evaporative cooling towards cool seasons is also supported by state-of-the-art Earth system models. Moreover, greening-triggered energy imbalance is propagated forward by atmospheric circulation to subsequent seasons and causes sizable time-lagged climate effects. Overall, greening makes winter warmer and summer cooler, attenuating the seasonal amplitude of NH temperature. These findings demonstrate complex tradeoffs and linkages of vegetation-climate feedbacks among seasons.

## Abstract (web summary)

The seasonal greening of Northern Hemisphere ecosystems due to extended growing periods and enhanced photosynthetic activity is, via experiments, shown to modify near-surface warming by perturbing land-atmosphere energy exchanges.

## Introduction

As air temperature rises, the phenological cycle of Northern Hemisphere (NH) ecosystems is shifting progressively towards earlier leaf emergence and delayed leaf senescence, which leads to rapid lengthening of the active growing season^1–4^. Remote sensing techniques have also clearly documented a widespread increase in leaf area and enhanced vegetation activity throughout the growing season, a phenomenon known as “vegetation greening”^5–8^. In turn, the altered phenology and structure of terrestrial plants could modulate the warming rate by changing the seasonal cycles of carbon, energy, and water at the land–atmosphere interface^4,9^. However, current knowledge about this climatic effect is limited mostly to enhanced carbon sequestration caused by a lengthened growing period and accelerated photosynthesis rates (biochemical feedbacks)^1,4,10^. The role of vegetation changes in the seasonal budget of surface energy fluxes (biophysical feedbacks)^11,12^ has been studied far less extensively, despite their strong capacity to affect regional to global warming over annual or longer timescales^13–15^.

Vegetation biophysics have long been recognized as a key regulator of seasonal air temperature climatology. For example, in early spring, the rate of air temperature increase rapidly decreases after leaf unfolding (typically for deciduous forests) due to increased transpiration after leaf-out that can effectively cool the leaf surface^12,16,17^. The critical role of plants in local temperature seasonality suggests that greening will alter the seasonality of NH warming at annual to decadal timescales. Vegetation greening also affects climate by interacting with other land-surface (e.g., snow or soil moisture) and atmospheric (e.g., water vapor, cloud, and circulation) processes^11,13,18^, the effects of which vary both geographically and seasonally^4^. For example, during snow-affected seasons/areas, vegetation greening markedly decreases snow cover and surface albedo due to a strong masking effect of darker canopies^11,18–20^, and the surface darkening would warm the surface that further accelerates snow losses^21,22^. In addition to affecting climate locally and transiently, vegetation biophysical processes summarized above can potentially trigger climate anomalies beyond the greening region (non-local feedbacks) and season (inter-seasonal feedbacks)^23–25^, by redistributing available energy over space and time. In particular, potential time-lagged temperature responses are hidden in those annually aggregated climatic consequences of greening^13^, and to some degree affect the seasonally asymmetric characteristics of NH warming^26–28^. However, our understanding of such effects and underlying processes remains rudimentary so far.

Observation-driven assessments compare the temperature difference between undisturbed forests and their neighboring non-forest counterparts as a proxy for temperature responses to forest gain, assuming the same climate background^14,29,30^. This space-for-time substitution is effective for quantifying localized and transient temperature feedbacks^14,29,30^, yet overlooks indirect atmospheric feedbacks due to, for example, water vapor, cloud, and circulation changes^31,32^. Hence, it is intrinsically flawed to detect non-local and/or inter-seasonal signals pertained to seasonal vegetation-climate feedbacks. Coupled land–atmosphere global climate models (GCM) offer a useful tool to detect causality in complex systems, e.g., the vegetation-climate interactions, though with uncertainties from a simplified representation of real-world processes^33^. GCMs link vegetation dynamics to climate changes by parameterizing the leaf area index (LAI) that determines land-surface boundary conditions and controls vegetation-atmospheric exchanges of water and energy^13,34^, and explicitly represent large-scale changes of atmospheric physical processes.

In this study, we use a series of simulations with the IPSL-CM (Institute Pierre Simon Laplace coupled model, see Methods) GCM^35^ to examine the biophysical effects of the observed widespread NH (defined here as 25–90°N) greening on seasonal warming patterns. Our key finding is that the cooling effect of NH greening is most prominent in summer, which, however, diminishes or even turns to net warming in cool seasons due to weakened evaporative capacity and enhanced albedo and water vapor feedbacks. We also show sizable time-lagged climate effects that greening in one season affects the temperature in the following ones by perturbing atmospheric circulation patterns. Such biophysical control of greening ultimately leads to a deviation of the seasonal warming trajectory from that originally driven by greenhouse warming.

## Results and discussion

### Coupled model experiments for detecting vegetation-climate feedback

We quantified changes of near-surface (2-m) air temperature (*T*_a_) in response to the observed NH greening for all active growing seasons during 1982–2014 using IPSL-CM. We defined the three growing seasons (spring, summer, and autumn) across the entire NH domain as periods of March-April-May (MAM), June-July-August (JJA), and September-October-November (SON), respectively. For each season, a pair of transient numerical experiments was performed by modifying LAI: a dynamic vegetation experiment (SCE) forced by annually and seasonally varying LAI from satellite observations^36^, and three seasonal control experiments (${{{{{{\rm{LAI}}}}}}}_{{{{{{\rm{CTL}}}}}}}^{{{{{{\rm{MAM}}}}}}}$, ${{{{{{\rm{LAI}}}}}}}_{{{{{{\rm{CTL}}}}}}}^{{{{{{\rm{JJA}}}}}}}$, and ${{{{{{\rm{LAI}}}}}}}_{{{{{{\rm{CTL}}}}}}}^{{{{{{\rm{SON}}}}}}}$ for MAM, JJA, and SON, respectively) forced by annually varying LAI for all seasons, except in the season of interest when the LAI was fixed to the climatological conditions observed during 1982–2014 (Fig. S1). For all experiments, other boundary conditions, including sea surface temperature (SST), sea ice fraction (SIC), and atmospheric CO_2_ concentrations, were kept consistent (Methods). Therefore, differences between SCE and the control experiments characterized the effects of the observed LAI changes on *T*_a_ (hereafter denoted as Δ*T*_a_), both intra- and inter-seasonally. Multimember paired ensembles were generated for each coupled model experiment by performing 30 repeated runs but with different initial conditions (see Methods).

The capacity of the IPSL-CM GCM for simulating the seasonal variations and spatial patterns of *T*_a_ was assessed by comparing the SCE simulation results with the observation-based *T*_a_ data (Methods). Throughout most of the growing season (May to October), the SCE simulation well reproduced the increasing trend and interannual variability of the NH land mean *T*_a_ observed during 1982–2014 (Fig. S2). Observational data showed that the strongest NH warming occurred in early spring (March and April) and late autumn (November). However, the SCE simulation failed to capture the exceptionally strong warming during the transitional seasons, leading to the underestimation of the annual mean warming trend (SCE: 0.237 ± 0.024 °C decade^−1^; observed: 0.362 ± 0.048 °C decade^−1^). This underestimation stemmed from a negative bias in the increase of downwelling shortwave radiation, possibly due to an absence of short-lived forcing and bias in the cloud systems^37^. Overall, the SCE reproduced the geographical patterns of seasonal warming reasonably well (Fig. S3), which strengthened our confidence in the model projections. Notably, it successfully captured the observed amplified warming over pan-arctic and semi-arid regions, as well as the few cases of regional cooling, such as that over northwestern North America during MAM (Fig. S3).

### Intra-seasonal temperature responses to NH LAI changes

**Fig. 1:**
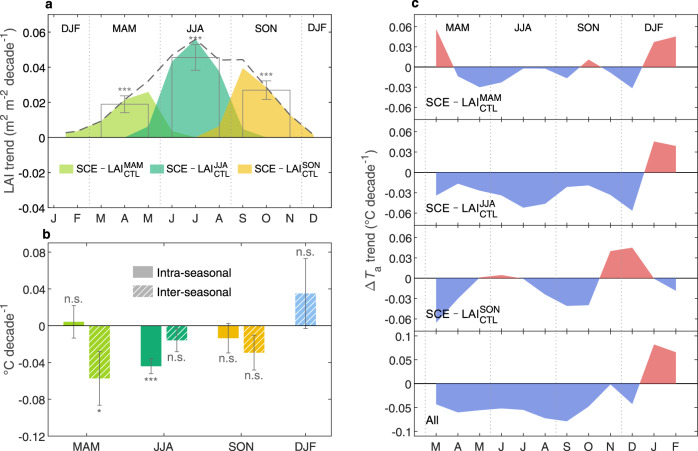
Intra- and inter-seasonal temperature responses to leaf area index (LAI) changes. **a** Monthly trends (shadings) of Northern Hemisphere (NH) mean LAI during 1982–2014 used as input to the seasonal simulations. The dashed curve and transparent bars indicate trends of monthly LAI and seasonally aggregated LAI values, respectively. **b** Linear trends of *T*_a_ driven by LAI changes within the same season (intra-seasonal) and other growing seasons (inter-seasonal). Error bars in **a**, **b** indicate uncertainty ranges [1 – standard deviation (SD)]. **c** Monthly trends of LAI-induced air temperature changes (Δ*T*_a_), with red and blue shadings representing positive and negative trends, respectively. The bottom panel shows the overall Δ*T*_a_ trends induced by LAI changes in all growing seasons, calculated as the sum of Δ*T*_a_ trends from the three seasonal runs shown separately in the above panels. ***p < 0.01; **p < 0.05; *p < 0.1; n.s., *p* > 0.1. MAM March-April-May, JJA June-July-August, SON September-October-November, DJF December-January-February.

For the period from 1982 to 2014, satellite-retrieved LAI showed statistically significant increasing trends (*p* < 0.05) during all growing seasons, with the most substantial increase observed in JJA (0.046 ± 0.007 m^2^ m^−2^ decade^−1^), followed by SON (0.027 ± 0.005 m^2^ m^−2^ decade^−1^) and MAM (0.019 ± 0.005 m^2^ m^−2^ decade^−1^)^5^ (Fig. 1a). Although forced identically by observed greening patterns, our GCM estimated highly divergent *T*_a_ responses across seasons, showing non-significant MAM warming (0.005 ± 0.018 °C decade^−1^, *p* > 0.1), strong and significant JJA cooling (−0.044 ± 0.008 °C decade^−1^, *p* < 0.05), and non-significant SON cooling (−0.014 ± 0.016 °C decade^−1^, *p* > 0.1) (intra-seasonal feedbacks shown in Fig. 1b). The LAI-induced JJA *T*_a_ trend was equivalent to cooling of −0.15 ± 0.03 °C in JJA over the study period, offsetting the overall SCE-simulated near-surface air warming over this period by ~12.5%. This strong JJA cooling was further supported by a significant negative correlation (*r* = −0.64, *p* < 0.05) between JJA LAI and its induced *T*_a_ anomalies (Fig. S4b), with greener summers (higher JJA LAI) co-occurring with more negative Δ*T*_a_. However, no such correlation was statistically detectable in MAM (*r* = −0.14, *p* > 0.1) or SON (*r* = 0.07, *p* > 0.1) (Fig. S4a, c), during which the LAI-induced changes accounted for only 1.3% (MAM) and −3.2% (SON) of the concurrent greenhouse warming. We also verified the robustness of our results by performing equilibrium experiments with an independent model, the NCAR Community Atmosphere Model coupled with Community Land Model (CAM-CLM, Methods). Indeed, this model generated a similarly strong LAI-induced cooling in JJA (−0.18 °C, *p* < 0.05 based on a two-sample *t*-test), which shifted to be much weaker in MAM (−0.02 °C, *p* > 0.1) and SON (−0.05 °C, *p* > 0.1) (Fig. S5).

**Fig. 2:**
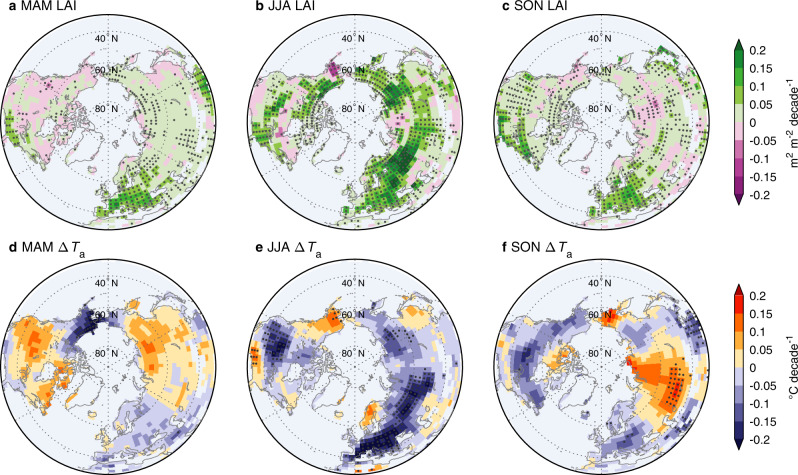
Spatial patterns of intra-seasonal temperature responses to leaf area index (LAI) changes. Maps of linear trends in seasonal LAI (**a**–**c**) and the induced air temperature changes (Δ*T*_a_) in the corresponding season (**d**–**f**) during 1982–2014. Δ*T*_a_ values reflect the differences in results between the dynamic vegetation experiment (SCE) and the three seasonal control experiments (${{{{{{\rm{LAI}}}}}}}_{{{{{{\rm{CTL}}}}}}}^{{{{{{\rm{MAM}}}}}}}$, ${{{{{{\rm{LAI}}}}}}}_{{{{{{\rm{CTL}}}}}}}^{{{{{{\rm{JJA}}}}}}}$, and ${{{{{{\rm{LAI}}}}}}}_{{{{{{\rm{CTL}}}}}}}^{{{{{{\rm{SON}}}}}}}$). Stippling indicates areas where the trend is statistically significant (*p* < 0.05). Pixels with climatological LAI < 0.1 are masked. MAM March-April-May, JJA June-July-August, SON September-October-November, DJF December-January-February.

Figure 2 shows the spatial distribution of the intra-seasonal *T*_a_ responses to LAI changes based on IPSL-CM. In the JJA experiment (SCE − ${{{{{{\rm{LAI}}}}}}}_{{{{{{\rm{CTL}}}}}}}^{{{{{{\rm{JJA}}}}}}}$), northern areas where Δ*T*_a_ decreased the most corresponded well to those where LAI showed the strongest greening, such as Europe, Russia, and the central U.S. (Fig. 2b, e). Similarly, increasing Δ*T*_a_ occurred in land areas that experienced the strongest browning, such as the southern U.S., Alaska, and semi-arid Asia (Fig. 2b, e). However, this spatial consistency of greening-cooling (or browning-warming) was not detected in most northern areas during MAM or SON (Fig. 2a, c, d, f). During these two seasons, though widespread areas showed significant greening (MAM: 42.0%; SON: 40.3%), only a small fraction (<5%) of northern lands exhibited significant trends of extra cooling or warming (Fig. 2a, c, d, f). In regions of Europe, East Asia. and the eastern U.S., the regional surface cooling in MAM and SON was also insignificant and discernably weaker than that in JJA, albeit with comparably strong greening signals across the three seasons. CAM-CLM also broadly agreed with the spatial patterns of signs despite varying magnitudes (Fig. S6). On one hand, these results imply a lower capacity for spring and autumn vegetation changes to influence near-surface climate, with potentially feedback processes different from summer periods. On the other hand, process-based uncertainties are more substantial in these relatively cold seasons associated with a model deficiency in representing vegetation–snow–moisture interactions.

### Inter-seasonal temperature responses to NH LAI changes

The IPSL-CM model also produced particularly strong time-lagged *T*_a_ responses to greening in previous growing seasons. For example, MAM greening-induced cooling in late spring carried over to early summer; JJA greening-induced cooling extended throughout the entire growing season, although cooling in autumn and spring of the next year was not as strong as that in summer; and SON LAI changes caused anomalously strong cooling in the following spring (Fig. 1c). In all growing seasons, NH greening consistently resulted in net warming during winter (December to February, DJF). For each focused season, the overall time-lagged response of *T*_a_ to previous-season greening was calculated by summing up the GCM-simulated *T*_a_ changes in response to greening in the two other growing seasons. At the scale of the NH, the GCM estimated Δ*T*_a_ trends of −0.057 ± 0.029 °C decade^−1^ (*p* = 0.06; MAM), −0.016 ± 0.013 °C decade^−1^ (*p* = 0.21; JJA), and −0.029 ± 0.019 °C decade^−1^ (*p* = 0.13; SON) in response to previous-season greening (Fig. 1b), corresponding to cooling of 0.19 ± 0.10 °C, 0.05 ± 0.04 °C, and 0.10 ± 0.06 °C, respectively during 1982−2014. In JJA, this cooling signal caused by previous greening amplified the transient cooling caused by JJA greening by ~36%. In MAM and SON, this inter-seasonal signal exceeded that caused by LAI changes within the same season (Fig. 1b), as also confirmed by the CAM-CLM results (Fig. S5), thereby dominating their overall *T*_a_ responses to LAI changes for the entire growing season. In both models, however, we did not detect the inter-seasonal signal with a very high statistical confidence level, due to trade-offs among regions and processes driving this carry-over effect.

**Fig. 3:**
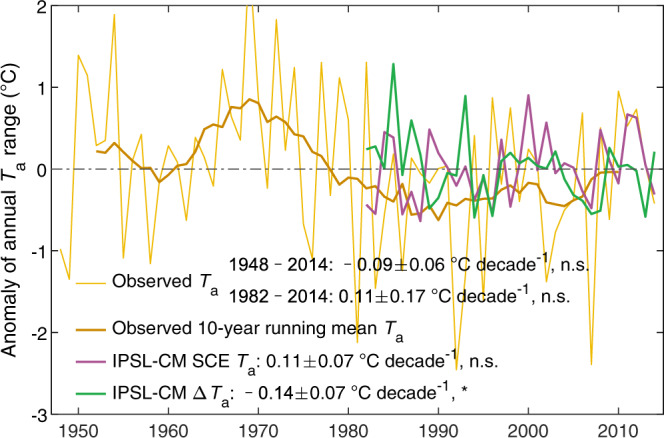
Vegetation-induced changes in the annual temperature range. Anomalies of observed (brown, from the Princeton Global Meteorological Forcing product) and IPSL-CM-simulated (purple, from the dynamic vegetation experiment SCE) annual ranges of Northern Hemisphere (NH) land mean air temperature (*T*_a_). The bold brown curve indicates the 10-year running mean of the observed annual *T*_a_ range. The green curve indicates the simulated annual *T*_a_ range induced by leaf area index (LAI) changes in all growing seasons combined. *p < 0.1; n.s., *p* > 0.1.

Examination of the intra- and inter-seasonal biophysical feedbacks together revealed that growing season vegetation greening had a discernable effect on the seasonal trajectory of the NH land mean *T*_a_ (Fig. 1c, bottom panel). Seasonal greening overall contributed to net warming in winter and net cooling throughout the entire growing season. By simultaneously causing warmer winters and cooler summers, NH greening reduced the amplitude of the *T*_a_ annual cycle (SAT, defined as July *T*_a_ minus January *T*_a_) at an average rate of −0.14 ± 0.07 °C decade^−1^ (*p* < 0.1) (Fig. 3), signaling to weaken *T*_a_ seasonality. Several previous studies reported that land SAT decreased throughout the twentieth century^26–28^. However, during the more recent 1982–2014 period, real-world observations and SCE results consistently showed a trend of leveling-off or a non-significant increase (+0.11 °C decade^−1^) (Fig. 3), indicating that greening diminished the recent increase in the seasonal amplitude of *T*_a_ in a warmer climate. In other words, in the absence of vegetation-climate feedbacks, northern lands would have experienced a much faster SAT increase, by about +0.25 °C decade^−1^, over the past 33 years.

### Driving processes of the seasonal vegetation-climate feedback

**Fig. 4:**
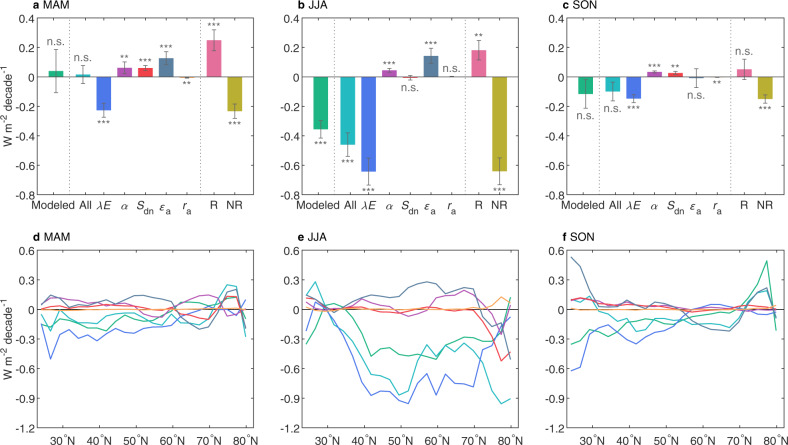
Biophysical processes underlying intra-seasonal temperature responses to greening. **a**–**c** Linear trends in surface radiative forcing associated with changes in latent heat flux (*λE*), surface albedo (*α*), aerodynamic resistance (*r*_a_), shortwave radiation (*S*_dn_), and air emissivity (${\varepsilon }_{{{{{{\rm{a}}}}}}}$) induced by leaf area index (LAI) changes within the same season. **d**–**f** Latitudinal variation in surface radiative forcing associated with different biophysical processes. “All” indicates the sum of all surface radiative forcings; “R” and “NR” indicate surface radiative forcing associated with radiative and non-radiative processes, respectively. ***p < 0.01; **p < 0.05; *p < 0.1; n.s., *p* > 0.1. MAM March-April-May, JJA June-July-August, SON September-October-November, DJF December-January-February.

An intriguing question is why the detected vegetation biophysical feedbacks on *T*_a_ vary in sign and magnitude among seasons. This variation is partly explained by the differing greening trends among seasons, with the most significant greening and cooling co-occurring in summer (Fig. 1a, b). Moreover, the major biophysical processes that govern the surface energy budget may also diverge seasonally. To fully understand the mechanisms of seasonal dependence among LAI-*T*_a_ feedbacks, we decomposed the modeled *T*_a_ response to LAI changes into contributions from different surface physical properties/processes, including surface albedo (*α*), evapotranspiration (*λE* or ET), surface incoming shortwave radiation (*S*_dn_), air longwave radiative emissivity (*ε*_a_), and aerodynamic resistance (*r*_a_) (see Methods). Together, these processes well reproduced the modeled LAI-induced net change in surface radiative forcing related to changes in *T*_a_ (Fig. 4a–c).

During all growing seasons, the ET-related change in surface radiative forcing was the greatest among the surface processes considered (Fig. 4a–c). The ET enhancement was paralleled with a proportional decrease in sensible heat (Fig. S7a–c). The less available surface energy dissipated as latent heat (Fig. S7d) favors the evaporative cooling of the surface. Geographically, regions with ET enhancement (Fig. 4d–f and S8b, h, n) also corresponded well to those of satellite-observed greening (Fig. 2a–c), confirming localized vegetation-temperature feedbacks through this key process^13,29^. Among the three growing seasons, greening generated a greater increase in the fraction of net solar radiation partitioned into ET (Fig. S7d), which resulted in a greater cooling in JJA (−0.64 ± 0.09 W m^−2^ decade^−1^, *p* < 0.05) than in MAM (−0.23 ± 0.05 W m^−2^ decade^−1^, *p* < 0.05) and SON (−0.15 ± 0.03 W m^−2^ decade^−1^, *p* < 0.05) (Fig. 4a–c). In JJA, plants often consume a greater amount of soil water to keep up with the greater water demand. For example, the ratio of transpiration to total ecosystem ET (T/ET)—a measure of vegetation control on surface climate through evaporative cooling^38^—was ~50% greater in JJA than in MAM/SON (Fig. S9a), which resulted in larger positive sensitivity of land ET to LAI translated to stronger surface cooling in JJA (Fig. S9a). The stronger evaporative cooling capacity of JJA greening than other growing seasons was also supported by 33 state-of-the-art Earth system models (Fig. S9b), suggesting that the seasonally varying LAI-*T*_a_ feedbacks are not dependent on our specific model use. Interestingly, within both MAM and SON, the LAI-*T*_a_ feedbacks shifted progressively from warming-dominated in colder months (i.e., March and November) to cooling-dominated in warmer months (i.e., May and September) (Fig. 1c). This phenomenon can be partly explained by the land–atmosphere theory that latent heat of vaporization is a more efficient cooling mechanism at higher temperatures^39^.

In addition to affecting how the land surface dissipates absorbed energy, greening also affects how much energy the surface absorbs (i.e., due to changes in *α*, *S*_dn_, and *ε*_a_). These radiative processes played a secondary, but often non-negligible, role, together increasing the surface radiative forcing at an average rate of 0.25 W m^−2^ decade^−1^ (*p* < 0.05; MAM), 0.18 W m^−2^ decade^−1^ (*p* < 0.05; JJA), and 0.05 W m^−2^ decade^−1^ (*p* < 0.05; SON) (Fig. 4a–c). During MAM when ET is constrained by relatively low temperatures, this radiative warming (as the combined effect of *α*, *S*_dn_, and *ε*_a_) overrode evaporative cooling and caused net surface warming (Fig. 4a). Increased *ε*_a_ contributed the most to this warming (Fig. 4a, d), as greening increased atmospheric water vapor content, which warmed the air by trapping extra longwave radiation^13,21^. Decreased α also significantly increased surface radiative forcing, particularly over greening and boreal/arctic regions, and throughout the seasonal cycle, such albedo-related warming peaked during MAM in the presence of snow cover^11,18^ (Fig. 4a, d and S8c). In JJA, radiative warming, as a result of decreased α and increased *ε*_a_, offset only 26% of evaporative cooling, causing net surface cooling. In SON, radiative warming due to decreased α and increased *S*_dn_ offset ~35% of the concurrent evaporative cooling, such that the surface cooling became insignificant.

**Fig. 5:**
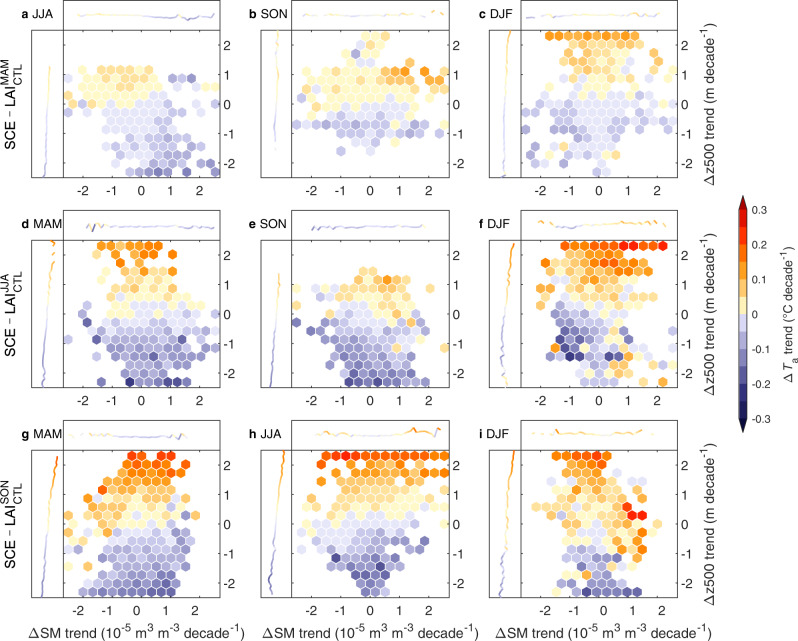
Biophysical processes underlying inter-seasonal temperature responses to greening. Distribution of the Δ*T*_a_ (air temperature) trend in the space of the ΔSM (soil moisture) trend and the Δz500 (500 hPa geopotential height) trend, all induced by MAM (**a**, **d**, **g**), JJA (**b**, **e**, **h**), and SON (**c**, **f**, **i**) leaf area index (LAI) changes. Marginal curves indicate the partial dependence of the Δ*T*_a_ trend on the ΔSM trend (top) and the Δz500 trend (left). MAM March-April-May, JJA June-July-August, SON September-October-November, DJF December-January-February.

We also evaluated how the widespread NH greening triggered inter-seasonal *T*_a_ responses. During those seasons when LAI was kept the same in the experiment pair (Table S1), any detected climate anomalies were linked to perturbations of soil and atmospheric conditions arising from LAI variations in the preceding season. At the NH scale, direct surface biophysical processes had poor explanatory power with respect to inter-seasonal changes in *T*_a_ (Fig. S10), signaling weak legacy effects through coupling between soil and surface boundary layer. However, regionally, soil moisture was a key factor connecting MAM LAI changes to JJA *T*_a_ responses, often showing regional warming in areas with soil drying (Figs. S11a, S12a). This occurred because MAM greening enhanced ET, causing a soil moisture deficit that was carried over to JJA; drier soils further enhanced JJA warming by dissipating absorbed solar radiation to a greater extent as sensible rather than latent heat^23^. In other cases, we found similar spatial patterns between trends of 500 hPa geopotential height (z500, an indicator of synoptic circulation changes impacting regional climate) and inter-seasonal Δ*T*_a_, with negative (positive) z500 trends corresponding to cooling (warming) (Figs. S11,S13). These patterns showed little similarity to greening patterns, suggesting that atmospheric circulation response connected the direct forcing of LAI changes on the local atmosphere to other regions and seasons. Via atmospheric teleconnections, SON NH greening triggered widespread MAM cooling centered in western Asia, as well as local MAM warming centered in northern Canada (Fig. S11g). This MAM cooling matched well with the SON LAI-induced MAM anomalous low, with the western Asia cooling center controlled by a cyclonic anomaly structure (Figs. S11g, S13g). By analyzing every repeated experiment with different initial conditions (Methods), we found similar teleconnection patterns in 19 out of 30 ensemble members (Fig. S14), suggesting that this pattern is more likely connected to vegetation greening than internal variability in the climate system. During DJF, an anomalous high prevailed over land forced by greening in all growing seasons (Fig. S13c, f, i), resulting in widespread NH warming (Fig. S11c, f, i), which implies that greening distributed more available solar energy toward the coolest periods of the seasonal cycle. By summarizing the Δ*T*_a_ responses in the space of soil moisture and z500 changes, we found that the inter-seasonal Δ*T*_a_ increases mostly followed a positive gradient of z500 trends (Fig. 5). Therefore, indirect atmospheric processes, such as warm/cold air mass advection and large-scale atmospheric circulation^25,40^, have played a key role in propagating the climatic effects of greening to other seasons.

In summary, this model-based study shows that LAI-*T*_a_ feedbacks through biophysical processes have apparent seasonal inconsistency regarding the signs and magnitudes, resulting from a trade-off between radiative warming and evaporative cooling. This is in contrast to the well-documented carbon-cycle feedbacks, for which NH greening exerts a seasonally consistent negative forcing on *T*_a_ through enhancing plant photosynthetic rates. Our results indicate that, in summer, the overwhelmingly strong ET enhancement forces net surface cooling, whereas in spring and autumn, radiative warming due to decreased albedo and higher atmospheric water vapor content nullify the evaporative cooling, producing weak and insignificant *T*_a_ changes. Compared with the straightforward response to greening, spring and autumn *T*_a_ changes are impacted to a greater extent by atmospheric circulation anomalies generated by greening in the preceding seasons. We however note potential uncertainties in our model-based LAI-*T*_a_ feedback results, particularly in terms of the inter-seasonal signal, for which solid observational constraints on the atmospheric circulation processes are presently lacking. We encourage other climate model centers to perform similar experiments to refine the exact values of vegetation biophysical feedbacks on seasonal *T*_a_ changes.

Our study achieves a holistic understanding of the seasonal responses of air temperature to NH greening over the growing season. We illustrate complex trade-offs and linkages of vegetation-climate feedbacks between different growing stages, which tend to be concealed within annually aggregated values^13^. This highlights the need to better understand these biophysical processes operating both within and across seasons so that their potential time-lagged climate benefits and/or counterproductive consequences will not be overlooked. The regulatory role of NH greening on seasonal climate also has implications for adaptation planning and decision-making, as greening is now increasingly shaped by human land-use practices such as afforestation and reforestation^41^.

## Materials and methods

### Transient experiments using coupled land–atmosphere models

We characterized vegetation-climate feedbacks using IPSL-CM, a coupled land–atmosphere GCM developed and maintained by the IPSL modeling community^42^. IPSL-CM has continuously contributed to the fifth and sixth phases of the Coupled Model Intercomparison Project (CMIP), which has informed assessment reports of the Intergovernmental Panel on Climate Change (IPCC)^43,44^. This GCM is composed of the Laboratoire de Météorologie Dynamique general circulation model with zooming capability (LMDZ)^45^ and the Organizing Carbon and Hydrology In Dynamic Ecosystems (ORCHIDEE)^46^ land surface model. Within the IPSL-CM coupled model, local exchanges of water and energy, as well as large-scale atmospheric circulation changes, are explicitly parameterized. The values of LAI and plant functional types (PFTs) modeled in ORCHIDEE were manually replaced with available satellite observations. Other vegetation-related biophysical parameters such as height, rooting depth, and leaf albedo were determined by prescribed PFT and a set of pre-defined PFT-specific values^46^. The module for calculating the surface energy budget was forced to use prescribed satellite observations, instead of model-simulated values, so observed vegetation changes could alter land-atmospheric energy fluxes and thus modulate *T*_a_.

Four GCM experiments were conducted to quantify the transient response of *T*_a_ to vegetation greening observed over each of the three growing seasons (MAM, JJA, and SON) during 1982–2014 (Table S1). The four experiments differed in the prescribed seasonal LAI conditions; the SCE run was forced by satellite-observed annually varying monthly LAI maps during 1982–2014, and the three control runs (${{{{{{\rm{LAI}}}}}}}_{{{{{{\rm{CTL}}}}}}}^{{{{{{\rm{MAM}}}}}}}$, ${{{{{{\rm{LAI}}}}}}}_{{{{{{\rm{CTL}}}}}}}^{{{{{{\rm{JJA}}}}}}}$, and ${{{{{{\rm{LAI}}}}}}}_{{{{{{\rm{CTL}}}}}}}^{{{{{{\rm{SON}}}}}}}$, corresponding to the three growing seasons) used the same LAI maps except for the season of interest, for which monthly varying climatological LAI maps for 1982–2014 were used. In each growing season, the difference between the experiment pair, with yearly varying and fixed present-season LAI (SCE–${{{{{{\rm{LAI}}}}}}}_{{{{{{\rm{CTL}}}}}}}^{{{{{{\rm{MAM}}}}}}}$ for MAM, SCE–${{{{{{\rm{LAI}}}}}}}_{{{{{{\rm{CTL}}}}}}}^{{{{{{\rm{JJA}}}}}}}$ for JJA, and SCE–${{{{{{\rm{LAI}}}}}}}_{{{{{{\rm{CTL}}}}}}}^{{{{{{\rm{SON}}}}}}}$ for SON), distinguished the biophysical feedbacks of observed seasonal LAI changes on the surface energy budget and *T*_a_ of both the present and subsequent seasons.

The satellite-observed LAI maps used for the experiments were obtained from the Global Inventory Monitoring and Modeling Studies (GIMMS) LAI3g, which are available at an 8-km spatial resolution and 15-day intervals for 1982–2014 (ref. ^36^). This global LAI product was generated from GIMMS AVHRR Normalized Difference Vegetation Index 3 g data using an artificial neural network algorithm^36^. The GIMMS LAI3g data replace possible snow- or cloud-contaminated LAI values with an average seasonal profile or interpolated values^36^. The high quality of this data product has been demonstrated through direct comparisons with field measurements^36^. This LAI product has been widely used to study the seasonal evolution of vegetation activity and its interactions with climate^23,37,47,48^. Moreover, other AVHRR-based LAI products (e.g., GLOBMAP^49^ and GLASS^50^) are available in the literature but have a considerable discrepancy regarding long-term LAI trends. Among these products, GIMMS LAI shows intermediate rates of greening seasonally^5^, thus offering a moderate estimate for LAI-climate feedback.

As satellite-based land cover maps are not available for the entire study period, we used the static 0.5-degree Olson land cover map generated by the ORCHIDEE community (https://forge.ipsl.jussieu.fr/orchidee/wiki/Documentation/Ancillary). The Olson map originally contains 94 land categories at 5 km resolution^51^, which were converted to the 12 PFTs and bare soil using a look-up table approach. The use of static PFT maps may to some degree bias the model results for regions where the LAI trends are dominated by human activities (e.g., agricultural intensification, plantation, deforestation), but will not have a hemispheric effect since the seasonal greening is contributed mainly by rising CO_2_ and climate change^5^. To meet the model input requirements, monthly LAI maps were first calculated as maximum values of 15-day LAI composites and then aggregated to 0.5° × 0.5° grid cells. Next, LAI maps at the PFT level were derived by weighting the satellite-observed LAI values by the fractional vegetation cover of each PFT within each grid cell.

Apart from seasonal LAI differences, all experiments were forced identically with realistic SST, SIC, and atmospheric CO_2_ concentrations observed from 1982–2014. The inclusion of observed surface boundary conditions allowed the GCM to better reproduce observed interannual and longer-term variations in global *T*_a_. Therefore, the separated vegetation-temperature feedbacks encompassed the indirect effects of vegetation–atmosphere–ocean interactions and CO_2_ radiative and physiological forcings, which are, in reality, embedded in observed temperature changes. Monthly SST and SIC maps were obtained from the Atmospheric Model Intercomparison Project (AMIP; http://www-pcmdi.llnl.gov/projects/amip/) with 1° × 1° spatial resolution. Observed global annual atmospheric CO_2_ concentrations were derived from those used for transient simulations in the “Trends and drivers of the regional scale sources and sink of carbon dioxide” (TRENDY) project (http://dgvm.ceh.ac.uk/node/9).

Figure S1 shows the steps for perfroming model simulations. During model spin-up, we performed 30-year undisturbed runs forced by climatological LAI, SST, SIC, and atmospheric CO_2_ to ensure that soil moisture and temperature reached an equilibrium with the climate conditions. Next, we extended the undisturbed runs for another 30 years to generate a 30-member initial-condition ensemble, with outputs of each year for providing an alternative initial condition. This approach is effective for minimizing uncertainties in modeled climate responses to perturbations related to internal climate variability^13,52^. Starting with these different initial conditions, we performed the four transient simulations using different LAI maps (Table S1) from January 1982 to December 2014 as input. We calculated the LAI-induced *T*_a_ changes (i.e., Δ*T*_a_) as the difference between SCE and the simulation with fixed seasonal LAI (${{{{{{\rm{LAI}}}}}}}_{{{{{{\rm{CTL}}}}}}}^{{{{{{\rm{MAM}}}}}}}$, ${{{{{{\rm{LAI}}}}}}}_{{{{{{\rm{CTL}}}}}}}^{{{{{{\rm{JJA}}}}}}}$, and ${{{{{{\rm{LAI}}}}}}}_{{{{{{\rm{CTL}}}}}}}^{{{{{{\rm{SON}}}}}}}$), both using the 30-member ensemble mean of the transient simulations. For each seasonal experiment, intra-seasonal *T*_a_ changes were quantified in the same season of fixed LAI, and inter-seasonal *T*_a_ changes (carry-over effects) were quantified for the remaining seasons. Only vegetated northern areas with climatological LAI ≥ 0.1 were considered in this analysis.

We also conducted numerical experiments using another coupled land–atmosphere model, CAM-CLM, to verify the robustness of our results. We used the model that couples the Community Atmosphere Model, version 6 (CAM6) and the Community Land Model, version 5 (CLM5)^53^, with a spatial resolution of 1.9° × 2.5°. Four 100-year-long equilibrium experiments were conducted with CAM-CLM using different LAI inputs: the LAI_2010s_ was prescribed with seasonally varying LAI averaged for the 2010–2014 period; and the three seasonal simulations (${{{{{{\rm{LAI}}}}}}}_{1980{{{{{\rm{s}}}}}}}^{{{{{{\rm{MAM}}}}}}}$, ${{{{{{\rm{LAI}}}}}}}_{1980{{{{{\rm{s}}}}}}}^{{{{{{\rm{JJA}}}}}}}$, and ${{{{{{\rm{LAI}}}}}}}_{1980{{{{{\rm{s}}}}}}}^{{{{{{\rm{SON}}}}}}}$) used the same LAI maps except for the focused season for which the average LAI for 1982–1986 was used (Table S2). The LAI-temperature feedbacks were quantified as the difference between the LAI_2010s_ and the seasonal simulations, averaged over the last 30 years of the 100-year-long runs.

### Observation-based *T*_a_ used for model evaluation

We used observation-based *T*_a_ data to evaluate the performance of the IPSL-CM GCM in simulating NH warming patterns and their seasonality. Air temperature data were derived from the Princeton Global Forcing (PGF) product developed by the Terrestrial Hydrology Research Group at Princeton University^54,55^. This forcing dataset provides near-surface meteorology data generated from the National Centers for Environmental Prediction-National Center for Atmospheric Research (NCEP-NCAR) reanalysis, which was bias-corrected based on the Climatic Research Unit (CRU) temperature^54^. The PGF temperature data are available at 3-h and 1° × 1° resolution covering the period 1948–2014.

### Analytical decomposition of LAI-induced *T*_a_ changes

We employed the analytical approaches of ref. ^13^ and ref. ^37^ to disentangle the contributions of key surface biophysical parameters to the modeled LAI-induced seasonal changes in surface radiative forcing. The considered biophysical processes and parameters included *λE* (latent heat flux, which cools the land surface by transferring absorbed energy to the troposphere), *α* (surface albedo, the ratio of reflected shortwave radiation to incoming shortwave radiation), *S*_dn_ (surface downward shortwave radiation, controlled by cloud and atmospheric water vapor content), *ε*_a_ (air emissivity, which reflects the effectiveness of air in emitting and absorbing longwave radiation from the surface), and *r*_a_ (aerodynamic resistance, which affects the efficiency of turbulent transfer of heat and water).

The decomposition was based inherently on the surface energy balance. Briefly, vegetation changes affect the surface energy budget and alter the *T*_s_, further warming or cooling the local near-surface air. The surface energy balance equation is written as:

1 $${S}_{{{{{{\rm{net}}}}}}}+{L}_{{{{{{\rm{net}}}}}}}=H+\lambda E+G$$

where *S*_net_, *L*_net_, *H*, and *G* are the surface net shortwave radiation, surface net longwave radiation, sensible heat flux, and ground heat flux, respectively. The left-hand side of Eq. (1) represents the net solar and thermal radiation absorbed by the surface (i.e., radiative processes), and the right-hand side represents the energy dissipation at the surface (i.e., non-radiative processes). *G* can be neglected on seasonal and interannual timescales, and the other terms can be expressed as follows:

2 $${S}_{{{{{{\rm{net}}}}}}}={S}_{{{{{{\rm{dn}}}}}}}-{S}_{{{{{{\rm{up}}}}}}}={S}_{{{{{{\rm{dn}}}}}}}\left(1-\alpha \right)$$

3 $$ {L}_{{{{{{\rm{net}}}}}}}={L}_{{{{{{\rm{dn}}}}}}}-{L}_{{{{{{\rm{up}}}}}}}={\varepsilon }_{{{{{{\rm{s}}}}}}}\sigma \left({\varepsilon }_{{{{{{\rm{a}}}}}}}{T}_{{{{{{\rm{a}}}}}}}^{4}-{T}_{{{{{{\rm{s}}}}}}}^{4}\right)$$

4 $$H=\rho {C}_{{{{{{\rm{p}}}}}}}\frac{{T}_{{{{{{\rm{s}}}}}}}-{T}_{{{{{{\rm{a}}}}}}}}{{r}_{{{{{{\rm{a}}}}}}}}$$

where *S*_dn_ and *S*_up_ are the surface downward and upward shortwave radiation, respectively, and *L*_dn_ and *L*_up_ are the surface downward and upward longwave radiation, respectively. Parameters *ε*_s_, *σ*, *ρ*, and *C*_p_ are the land surface emissivity (0.95), Stephan–Boltzmann constant (5.67 × 10^−8^ W m^−2^ K^−4^), air density (1.205 kg m^−3^), and specific heat capacity of the air at constant pressure (1013 J kg^−1^ K^−1^), respectively.

By combining Eqs. (2–4) and differentiating Eq. (1) with respect to *T*_s_, Δ*T*_s_ can be derived as follows:

5 $$\Delta {T}_{{{{{{\rm{s}}}}}}}=\;	 \frac{1}{{f}_{{{{{{\rm{s}}}}}}}}\left(-{S}_{{{{{{\rm{dn}}}}}}}\Delta \alpha +\left(1-\alpha \right){\Delta S}_{{{{{{\rm{dn}}}}}}}-\Delta \lambda E+\frac{\rho {C}_{{{{{{\rm{p}}}}}}}}{{r}_{{{{{{\rm{a}}}}}}}^{2}}\left({T}_{{{{{{\rm{s}}}}}}}-{T}_{{{{{{\rm{a}}}}}}}\right){\Delta r}_{{{{{{\rm{a}}}}}}}+{\varepsilon }_{{{{{{\rm{s}}}}}}}\sigma {T}_{{{{{{\rm{a}}}}}}}^{4}{\Delta \varepsilon }_{{{{{{\rm{a}}}}}}}\right)\\ 	 +\left(\frac{\rho {C}_{{{{{{\rm{p}}}}}}}}{{r}_{{{{{{\rm{a}}}}}}}}+{4\varepsilon }_{{{{{{\rm{s}}}}}}}\sigma {\varepsilon }_{{{{{{\rm{a}}}}}}}{T}_{{{{{{\rm{a}}}}}}}^{3}\right)\Delta {T}_{{{{{{\rm{a}}}}}}}/\left(\frac{\rho {C}_{{{{{{\rm{p}}}}}}}}{{r}_{{{{{{\rm{a}}}}}}}}+{4\varepsilon }_{{{{{{\rm{s}}}}}}}\sigma {T}_{{{{{{\rm{s}}}}}}}^{3}\right)$$

6 $${f}_{{{{{{\rm{s}}}}}}}=\frac{\rho {C}_{{{{{{\rm{p}}}}}}}}{{r}_{{{{{{\rm{a}}}}}}}}+{4\varepsilon }_{{{{{{\rm{s}}}}}}}\sigma {T}_{{{{{{\rm{s}}}}}}}^{3}$$

where *f*_s_ is the surface energy redistribution factor, which represents changes in *T*_s_ in response to radiative forcing of 1 W m^−2^ at the land surface. The first term on the right-hand side of Eq. (5) denotes LAI-induced *T*_s_ changes due to altered surface radiative forcing ($\Delta {T}_{{{{{{\rm{s}}}}}}}^{{{{{{\rm{rad}}}}}}}={f}_{s}^{-1}(-{S}_{{{{{{\rm{dn}}}}}}}\Delta \alpha +\left(1-\alpha \right){\Delta S}_{{{{{{\rm{dn}}}}}}}-\Delta \lambda E+\rho {C}_{{{{{{\rm{p}}}}}}}\left({T}_{{{{{{\rm{s}}}}}}}-{T}_{{{{{{\rm{a}}}}}}}\right){r}_{{{{{{\rm{a}}}}}}}^{-2}{\Delta r}_{{{{{{\rm{a}}}}}}}+{\varepsilon }_{{{{{{\rm{s}}}}}}}\sigma {T}_{{{{{{\rm{a}}}}}}}^{4}{\Delta \varepsilon }_{{{{{{\rm{a}}}}}}})$, and the second term demonstrates that LAI-induced *T*_s_ changes can be almost equivalently converted to *T*_a_ changes ($\Delta {T}_{{{{{{\rm{a}}}}}}}^{{{{{{\rm{rad}}}}}}}=(\rho {C}_{{{{{{\rm{p}}}}}}}{r}_{{{{{{\rm{a}}}}}}}^{-1}+{4\varepsilon }_{{{{{{\rm{s}}}}}}}\sigma {T}_{{{{{{\rm{s}}}}}}}^{3})\Delta {T}_{{{{{{\rm{s}}}}}}}^{{{{{{\rm{rad}}}}}}}/(\rho {C}_{{{{{{\rm{p}}}}}}}{r}_{{{{{{\rm{a}}}}}}}^{-1}+{4\varepsilon }_{{{{{{\rm{s}}}}}}}\sigma {\varepsilon }_{{{{{{\rm{a}}}}}}}{T}_{{{{{{\rm{s}}}}}}}^{3})$). Local Δ*T*_a_ changes unexplained by direct surface-atmospheric energy exchange ($\Delta {T}_{{{{{{\rm{a}}}}}}}^{{{{{{\rm{rad}}}}}}}$) can be attributed to air mass advection or changes in atmospheric circulation ($\Delta {T}_{{{{{{\rm{a}}}}}}}^{{{{{{\rm{cir}}}}}}}$), as follows:

7 $$\Delta {T}_{{{{{{\rm{a}}}}}}}=\frac{1}{{f}_{{{{{{\rm{a}}}}}}}}\left(-{S}_{{{{{{\rm{dn}}}}}}}\Delta \alpha +\left(1-\alpha \right){\Delta S}_{{{{{{\rm{dn}}}}}}}-\Delta \lambda E+\frac{\rho {C}_{{{{{{\rm{p}}}}}}}}{{r}_{{{{{{\rm{a}}}}}}}^{2}}\left({T}_{{{{{{\rm{s}}}}}}}-{T}_{{{{{{\rm{a}}}}}}}\right){\Delta r}_{{{{{{\rm{a}}}}}}}+{\varepsilon }_{{{{{{\rm{s}}}}}}}\sigma {T}_{{{{{{\rm{a}}}}}}}^{4}{\Delta \varepsilon }_{{{{{{\rm{a}}}}}}}\right)+\Delta {T}_{{{{{{\rm{a}}}}}}}^{{{{{{\rm{cir}}}}}}}$$

where *f*_a_ is the surface energy redistribution factor for *T*_a_, given by

8 $${f}_{{{{{{\rm{a}}}}}}}=\frac{\rho {C}_{{{{{{\rm{p}}}}}}}}{{r}_{{{{{{\rm{a}}}}}}}}+{4\varepsilon }_{{{{{{\rm{s}}}}}}}\sigma {\varepsilon }_{{{{{{\rm{a}}}}}}}{T}_{{{{{{\rm{s}}}}}}}^{3}$$

Similar to previous assessments^13,37^, climatic effects of vegetation changes associated with different biophysical processes were assessed with equivalent surface radiative forcing, to ensure direct comparability among them. In Eq. (7), $-{S}_{{{{{{\rm{dn}}}}}}}\Delta \alpha$, $\left(1-\alpha \right){\Delta S}_{{{{{{\rm{dn}}}}}}}$, $-\Delta \lambda E$, $\rho {C}_{{{{{{\rm{p}}}}}}}{r}_{{{{{{\rm{a}}}}}}}^{-2}\left({T}_{{{{{{\rm{s}}}}}}}-{T}_{{{{{{\rm{a}}}}}}}\right){\Delta r}_{{{{{{\rm{a}}}}}}}$, and ${\varepsilon }_{{{{{{\rm{s}}}}}}}\sigma {T}_{{{{{{\rm{a}}}}}}}^{4}{\Delta \varepsilon }_{{{{{{\rm{a}}}}}}}$ represent surface radiative forcing caused by vegetation-induced changes of *α*, *S*_dn_, *λE*, *r*_a_, and *ε*_a_, respectively. The sum of$-{S}_{{{{{{\rm{dn}}}}}}}\Delta \alpha$, $\left(1-\alpha \right){\Delta S}_{{{{{{\rm{dn}}}}}}}$, $-\Delta \lambda E$ and ${\varepsilon }_{{{{{{\rm{s}}}}}}}\sigma {T}_{{{{{{\rm{a}}}}}}}^{4}{\Delta \varepsilon }_{{{{{{\rm{a}}}}}}}$ represents radiative forcing associated with radiative processes, and the sum of $-\Delta \lambda E$ and $\rho {C}_{{{{{{\rm{p}}}}}}}{r}_{{{{{{\rm{a}}}}}}}^{-2}\left({T}_{{{{{{\rm{s}}}}}}}-{T}_{{{{{{\rm{a}}}}}}}\right){\Delta r}_{{{{{{\rm{a}}}}}}}$ represents radiative forcing associated with non-radiative processes.

## Supplementary information

Supplementary Information

## Data Availability

All observation and model data that support the findings of this study are available as follows. The AVHRR GIMMS LAI3g data were available at http://cliveg.bu.edu/modismisr/lai3g-fpar3g.html. The climatic variables from the Princeton Global Meteorological Forcing (PGF) v3.0 data were available at https://hydrology.princeton.edu/data.pgf.php/.

## Code availability

Code and documentation for the IPSL model are publicly available at https://cmc.ipsl.fr/ipsl-climate-models/ipsl-cm5/. Description and code relevant to CAM6 and CLM5 are freely available online at https://www.cesm.ucar.edu:/models/cesm2. Model outputs were processed using the software Matlab 2019a. The MATLAB scripts are available from the corresponding author upon request.
