# Supplementary material for: Biophysical impacts of northern vegetation changes on seasonal warming patterns
Source: Nat Commun. 2022 Jul 7;13:3925. doi: 10.1038/s41467-022-31671-z (PMC9262912; doi:10.1038/s41467-022-31671-z)
Supplement: Supplementary file 1 — Supplementary Information [file 41467_2022_31671_MOESM1_ESM.pdf]

Supplementary Information for

Biophysical impacts of northern vegetation changes on seasonal warming patterns

by Lian *et al.*

This PDF file contains:

Supplementary Tables 1-2

Supplementary Figures 1-14

Supplementary Tables

Supplementary Table 1. Experimental design for quantifying the climate feedbacks of seasonal LAI changes in this study. Here, "variable LAI" means that the simulation was forced with observed annually varying LAI values (from GIMMS LAI 3g) over the 1982−2014 period for the corresponding season, and "fixed LAI" means that climatological mean LAI values over 1982−2014 were used. Similarly, "variable SST/SIC/CO_2" refers to the use of annually varying sea-surface temperature, sea-ice fraction, or atmospheric CO_2 concentration observed over 1982−2014.

Simulations	MAM LAI	JJA LAI	SON LAI	DJF LAI	SST/SIC (all months)	Atmospheric CO_2 (all months)
SCE	Variable	Variable	Variable	Variable	Variable	Variable
LAI_{CTL}^{MAM}	**Fixed**	Variable	Variable	Variable	Variable	Variable
LAI_{CTL}^{JJA}	Variable	**Fixed**	Variable	Variable	Variable	Variable
LAI_{CTL}^{SON}	Variable	Variable	**Fixed**	Variable	Variable	Variable

Supplementary Table 2. Experimental design for the CAM-CLM model. Here, "1980s", "2010s" and "Climatology" mean that the simulation was forced with observed LAI values averaged for the corresponding season over 1982−1986, 2010−2014, and the entire 1982−2014 period, respectively.

Simulations	MAM LAI	JJA LAI	SON LAI	DJF LAI	SST/SIC (all months)	Atmospheric CO_2 (all months)
LAI_{2010s}	2010s	2010s	2010s	2010s	Climatology	Climatology
LAI_{1980s}^{MAM}	**1980s**	2010s	2010s	2010s	Climatology	Climatology
LAI_{1980s}^{JJA}	2010s	**1980s**	2010s	2010s	Climatology	Climatology
LAI_{1980s}^{SON}	2010s	2010s	**1980s**	2010s	Climatology	Climatology

Supplementary Figures

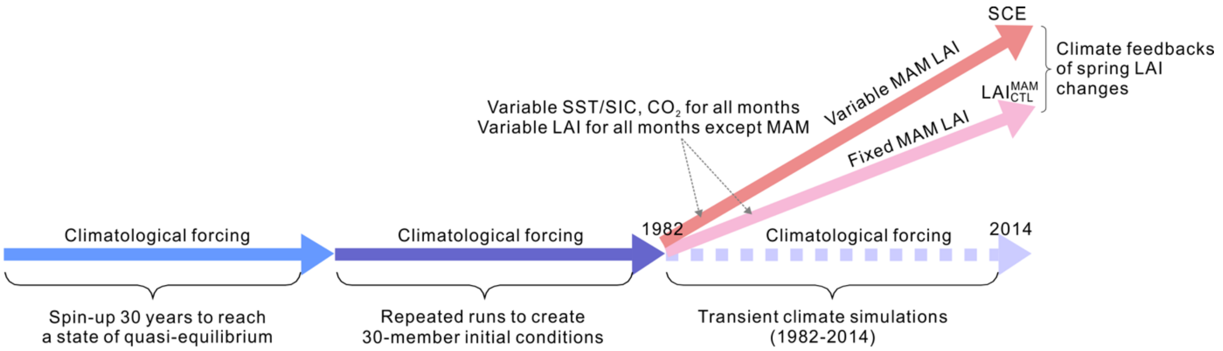

Supplementary Figure 1. Design of transient experiments for quantifying the climate feedbacks of seasonal LAI changes using the experimental design for MAM as an example. See details of the procedures in Methods.

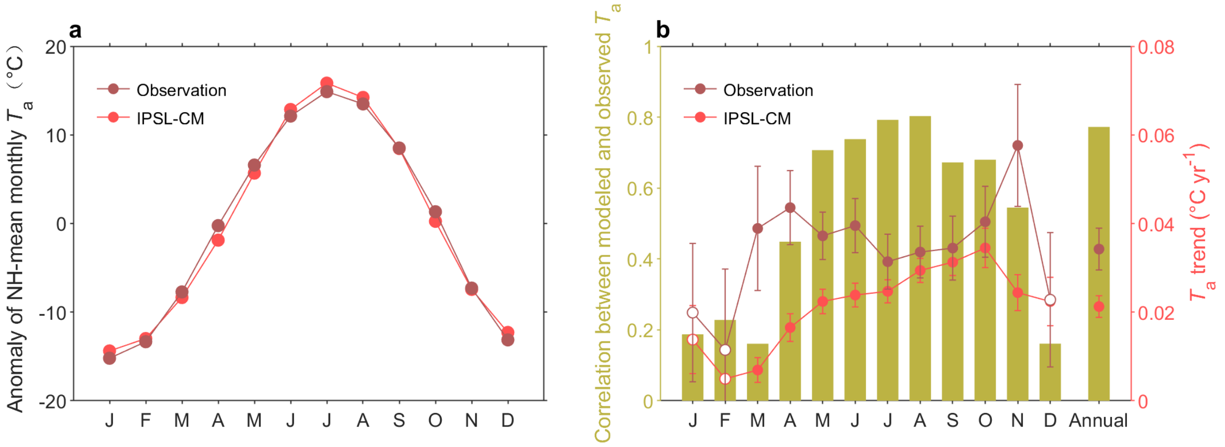

Supplementary Figure 2. IPSL-simulated versus observed NH-mean T_a. (**a**) Simulated and observed climatological monthly T_a anomalies (minus the multi-year annual average T_a over 1982–2014). (**b**) Monthly trends of simulated and observed time series of T_a anomalies (curves) and Pearson correlation coefficients between the time series (brown bars) for both the annual mean and individual months. Closed and open dots indicate $p < 0.05$ and $p > 0.05$, respectively. Error bars indicate uncertainty ranges [1 – standard deviation (SD)]. The observed T_a is from the Princeton Global Meteorological Forcing (PGF) data, and the simulated T_a is from the SCE experiment (Methods).

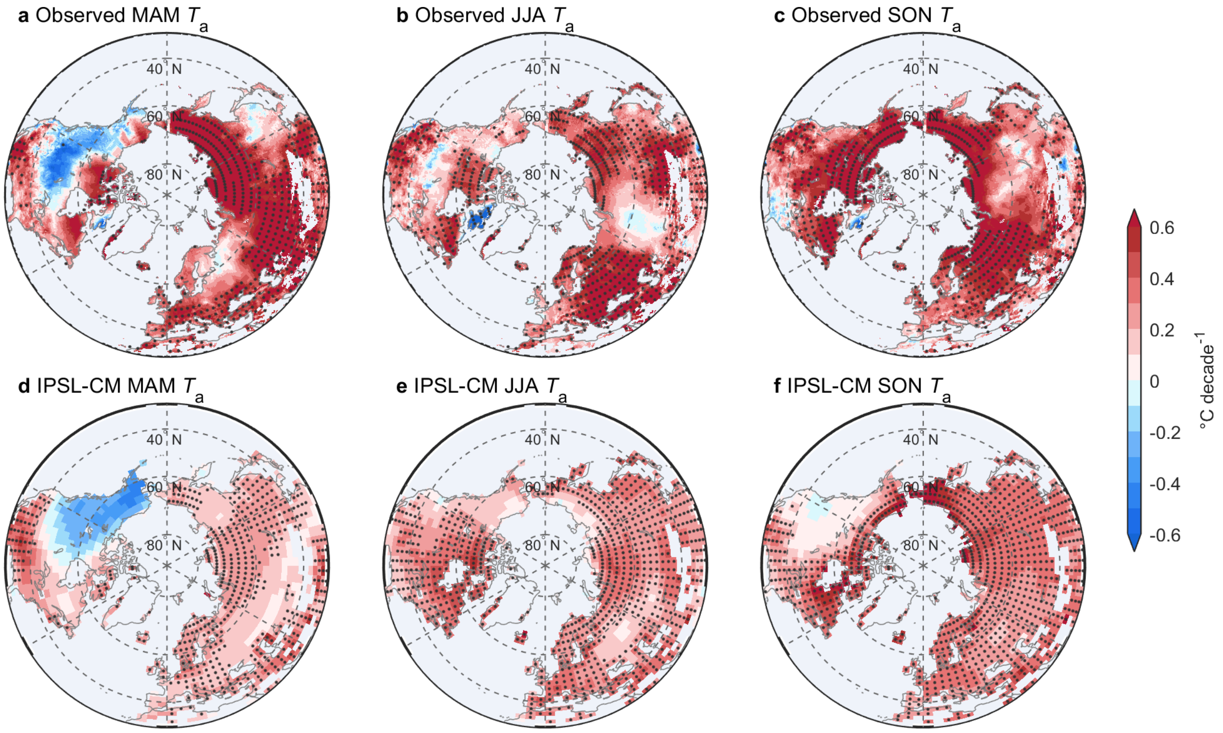

Supplementary Figure 3. Spatial patterns of the IPSL-simulated versus observed seasonal trends of T_a for 1982−2014. Pixels with climatological LAI < 0.1 are masked out. Stippling indicates areas where the trend is statistically significant ($p < 0.05$). The observed T_a is from the Princeton Global Meteorological Forcing (PGF) data, and the simulated T_a is from the SCE experiment (Methods).

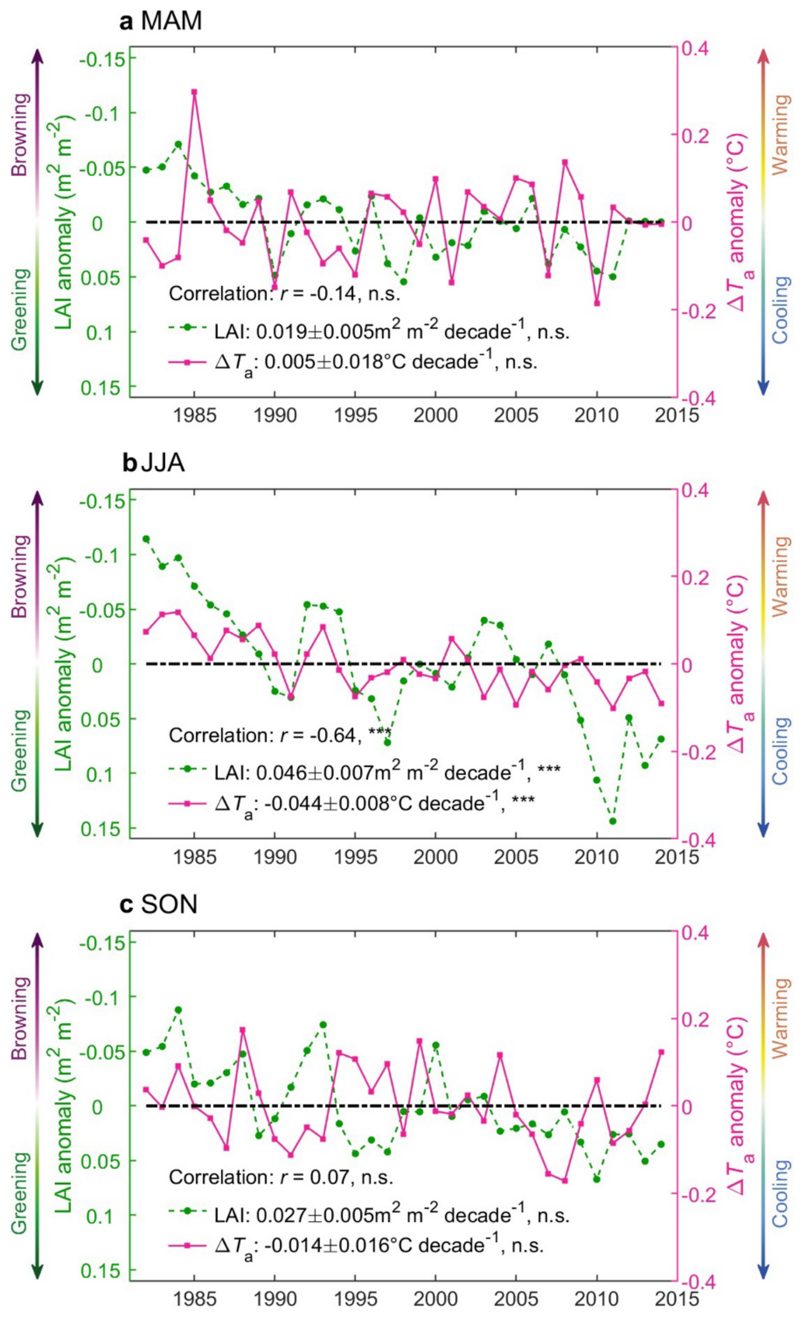

Supplementary Figure 4. Anomalies of seasonal NH-mean LAI and its induced T_a changes (ΔT_a) for the same season. Labels indicate the correlation between the LAI and ΔT_a anomalies, and their respective linear trends. ***$p < 0.01$; **$p < 0.05$; *$p < 0.1$; n.s., $p > 0.1$.

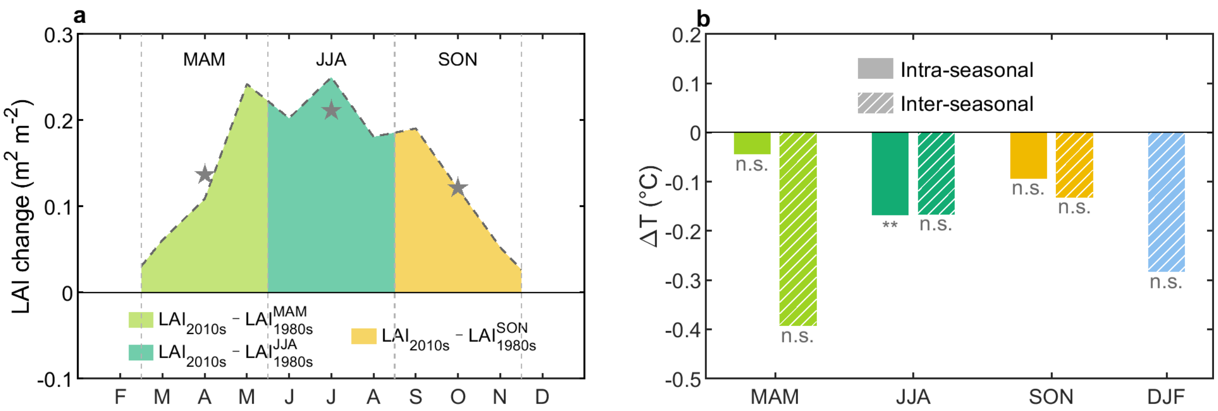

Supplementary Figure 5. Intra- and inter-seasonal temperature responses to LAI changes based on CAM-CLM. a, Shadings show the LAI changes of the three growing seasons from 1982−1986 to 2010-2014, used as input for the seasonal simulations, respectively. Pentagrams indicate seasonally aggregated values of the LAI change. **b**, LAI-induced T_a changes (ΔT_a) within the same season (intra-seasonal) and other growing seasons (inter-seasonal). ** (n.s.) indicates the difference is (in)significant at the 95% confidence interval based on a two-sample t test.

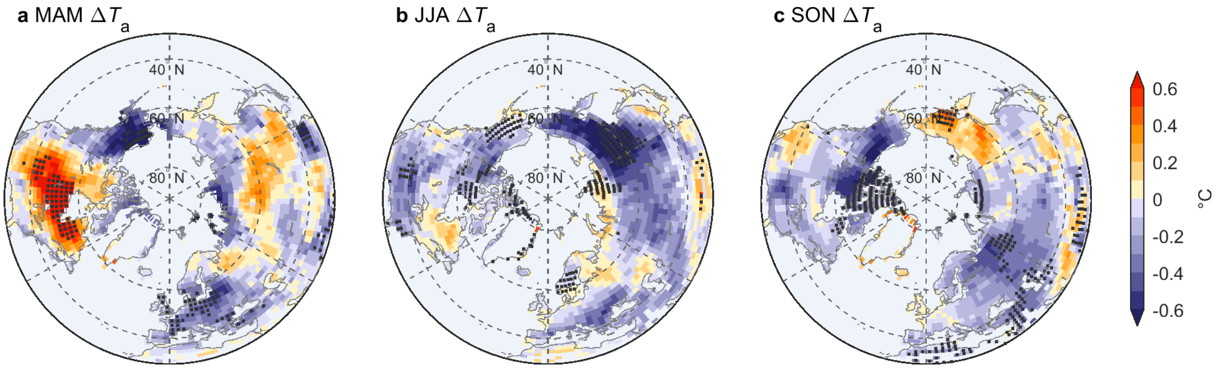

Supplementary Figure 6. Spatial patterns of intra-seasonal temperature responses to LAI changes based on CAM-CLM. Maps of MAM (**a**), JJA (**b**) and SON (**c**) T_a changes (ΔT_a) (**d–f**) from 1982−1986 to 2010-2014, in response to LAI changes of same season. ΔT_a was calculated the differences in results between LAI_{2010s} and seasonal runs (LAI_{1980s}^{MAM}, LAI_{1980s}^{JJA} and LAI_{1980s}^{SON}), averaged for the last 30 years. Pixels with climatological LAI < 0.1 are masked. Stippling indicates the difference is significant at the 95% confidence interval based on a two-sample *t* test.

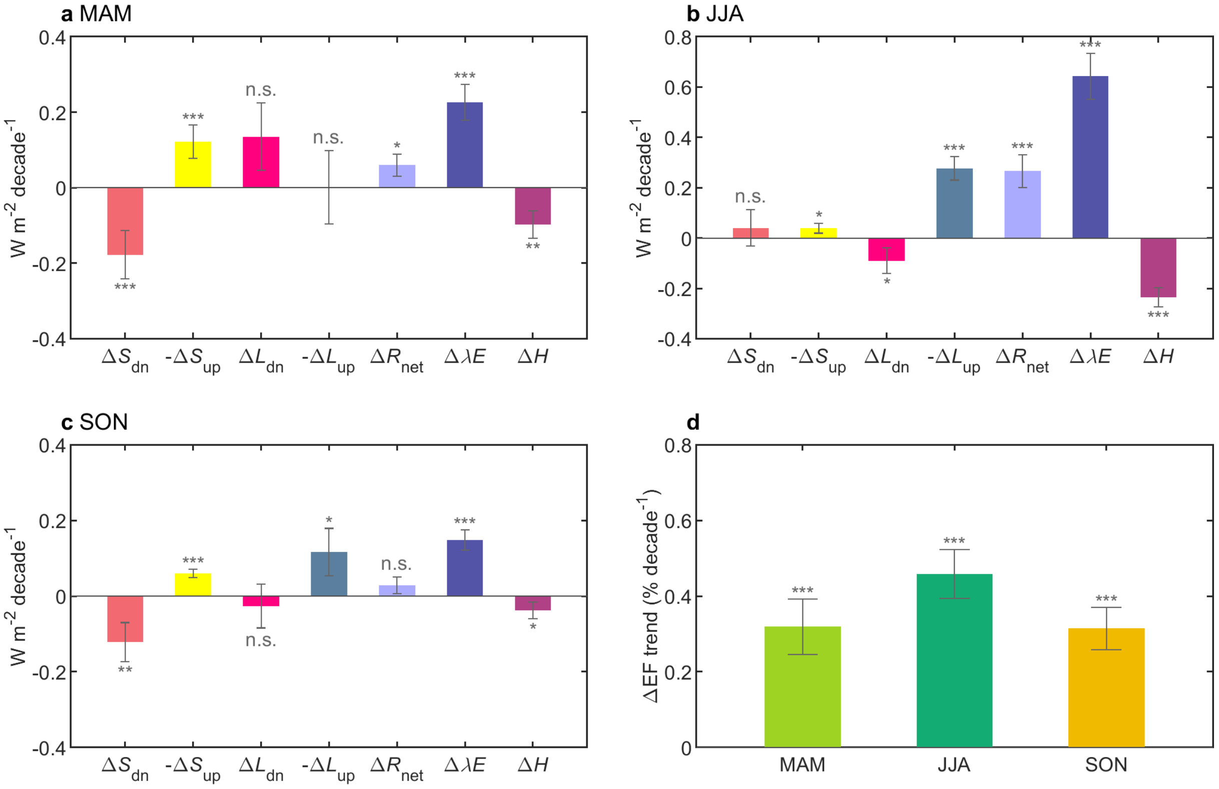

Supplementary Figure 7. LAI-induced changes in surface energy fluxes. a–c, Linear trends in LAI-induced changes of surface energy fluxes for the same season, including downward (S_{dn}) and upward (S_{up}) shortwave radiation, downward (L_{dn}) and upward (L_{up}) longwave radiation, net solar radiation (R_{net}), latent heat (λE), and sensible heat (H). Note that opposite numbers of ΔS_{up} and ΔL_{up} are used to represent positive radiative forcing with positive values. **d**, Linear trends in evaporative fraction (EF, the fraction of λE to R_{net}) induced by LAI changes within the same season. ***$p < 0.01$; **$p < 0.05$; *$p < 0.1$; n.s., $p > 0.1$.

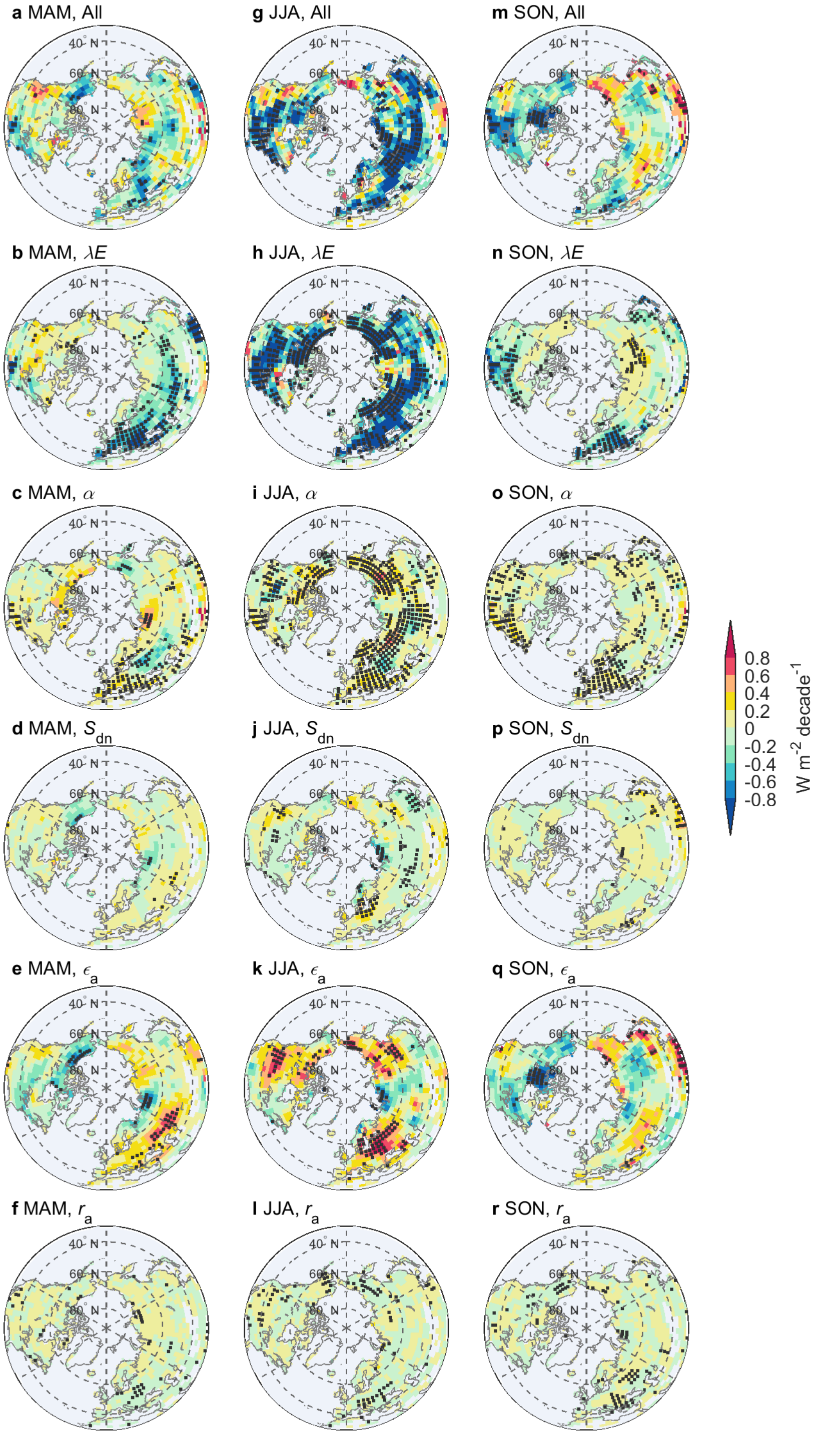

Supplementary Figure 8. Spatial patterns of the linear trends in surface radiative forcing associated with changes in latent heat (λE; **b**, **h**, **n**), surface albedo (α; **c**, **i**, **o**), aerodynamic resistance (r_a; **f**, **l**, **r**), downward shortwave radiation (S_{dn}; **d**, **j**, **p**), and air emissivity (ε_a; **e**, **k**, **q**) induced by LAI changes of the same season. "All" represents the sum of all surface radiative forcing. Stippling indicates areas where the trend is statistically significant ($p < 0.05$).

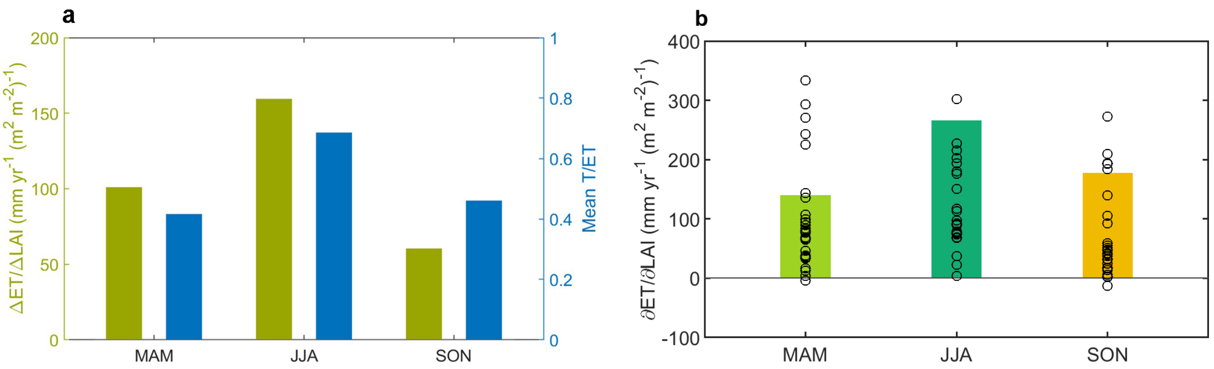

Supplementary Figure 9. a, IPSL-simulated NH mean T/ET ratio (the ratio of plant transpiration to total terrestrial ET, blue bars) and ΔET/ΔLAI (the sensitivity of land ET to LAI increase, green bars) over 1982−2014 for different growing seasons. This evaluation was based on outputs from the IPSL-CM SCE run. **b**, Seasonal values of ∂ET/∂LAI (the partial sensitivity of ET to LAI) over 1982−2014, for both individual models (circles) and the multi-model mean (bars). This analysis was based on outputs from 33 Earth system models archived in Coupled Model Intercomparison Project 6 (CMIP6), including ACCESS-ESM1-5, AWI-ESM-1-1-LR, BCC-CSM2-MR, BCC-ESM1, CESM2, CESM2-FV2, CESM2-WACCM, CESM2-WACCM-FV2, CMCC-CM2-SR5, CMCC-ESM2, CanESM5, E3SM-1-0, E3SM-1-1, E3SM-1-1-ECA, EC-Earth3-CC, EC-Earth3-Veg, EC-Earth3-Veg-LR, FGOALS-g3, FIO-ESM-2-0, GFDL-ESM4, GISS-E2-1-G, GISS-E2-1-H, INM-CM5-0, IPSL-CM5A2-INCA, IPSL-CM6A-LR, IPSL-CM6A-LR-INCA, MPI-ESM-1-2-HAM, MPI-ESM1-2-HR, MPI-ESM1-2-LR, NorESM2-LM, NorESM2-MM, SAM0-UNICON and TaiESM1. The ∂ET/∂LAI is calculated through a multi-variate linear regression of ET against LAI, air temperature, precipitation and shortwave solar radiation.

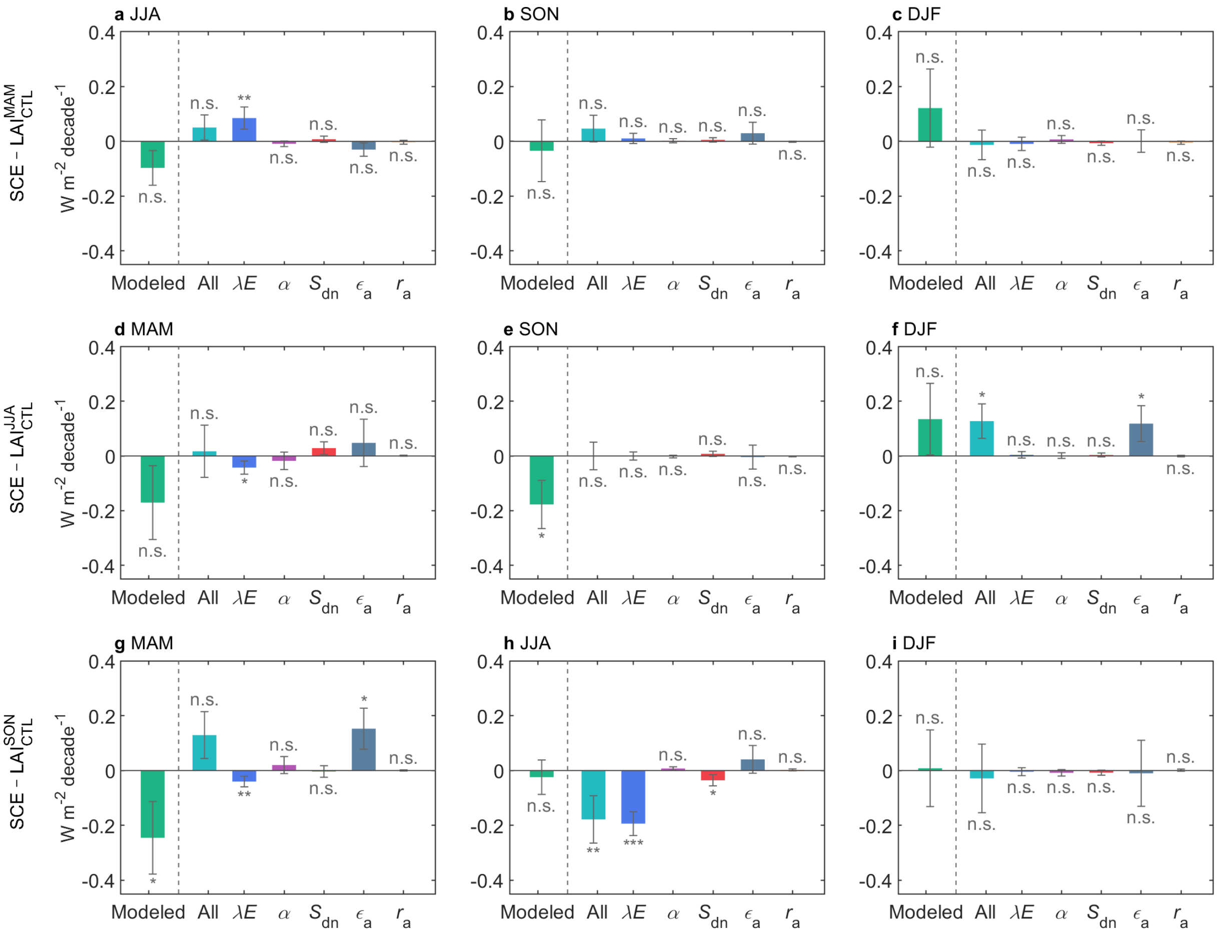

Supplementary Figure 10. Linear trends in surface radiative forcing associated with changes in latent heat (λE), surface albedo (α), aerodynamic resistance (r_a), shortwave radiation (S_{dn}), and air emissivity (ε_a) induced by concurrent changes of MAM (**a-c**), JJA (**d-f**), and SON (**g-i**) LAI over 1982−2014. "All" represents the arithmetic sum of all surface radiative forcing. ***$p < 0.01$; **$p < 0.05$; *$p < 0.1$; n.s., $p > 0.1$.

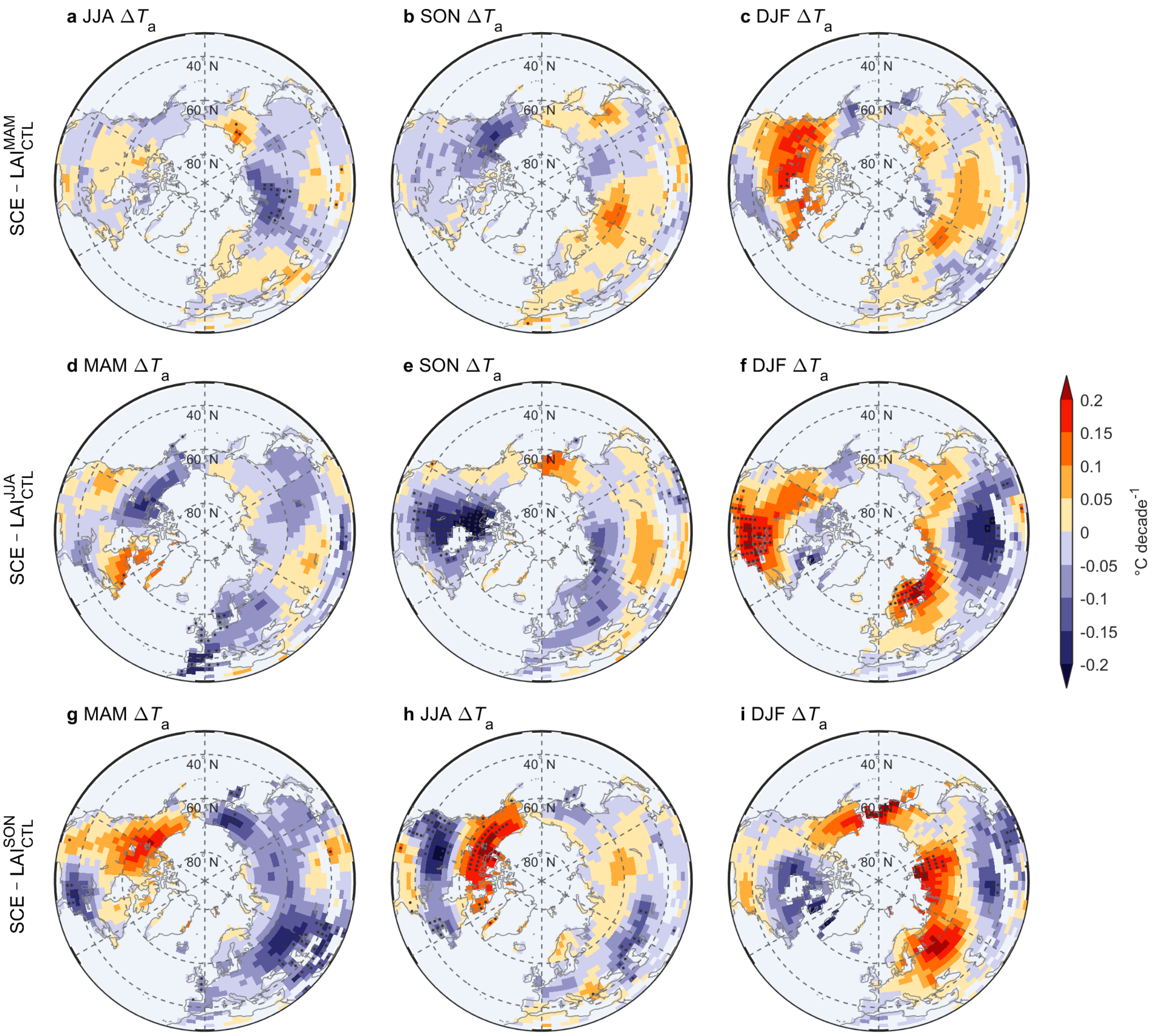

Supplementary Figure 11. Spatial patterns of inter-seasonal temperature responses to LAI changes. Maps show the linear trends of T_a induced by MAM (**a-c**), JJA (**d-f**), and SON (**g-i**) LAI changes over 1982−2014. The ΔT_a was calculated as the difference between SCE and the simulation with fixed seasonal LAI (LAI_{CTL}^{MAM} , LAI_{CTL}^{JJA} , and LAI_{CTL}^{SON}). Pixels with climatological LAI < 0.1 are masked out. Stippling indicates areas where the trend is statistically significant ($p < 0.05$).

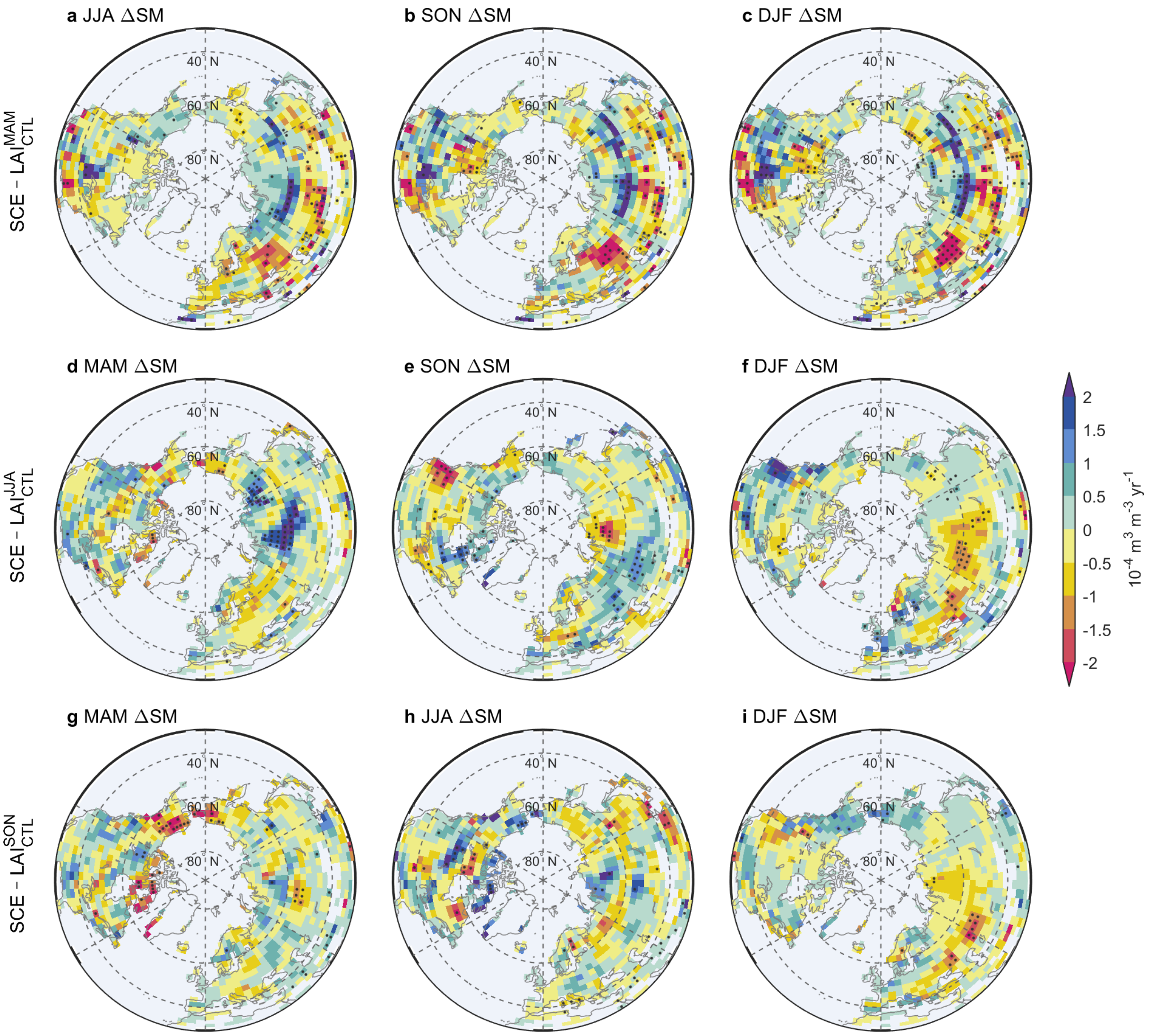

Supplementary Figure 12. Spatial patterns of inter-seasonal soil moisture (SM) responses to LAI changes. Maps show the linear trends of SM induced by MAM (**a-c**), JJA (**d-f**), and SON (**g-i**) LAI changes over 1982−2014. The ΔSM was calculated as the difference between SCE and the simulation with fixed seasonal LAI (LAI_{CTL}^{MAM}, LAI_{CTL}^{JJA}, and LAI_{CTL}^{SON}). Pixels with climatological LAI < 0.1 are masked out. Stippling indicates areas where the trend is statistically significant ($p < 0.05$).

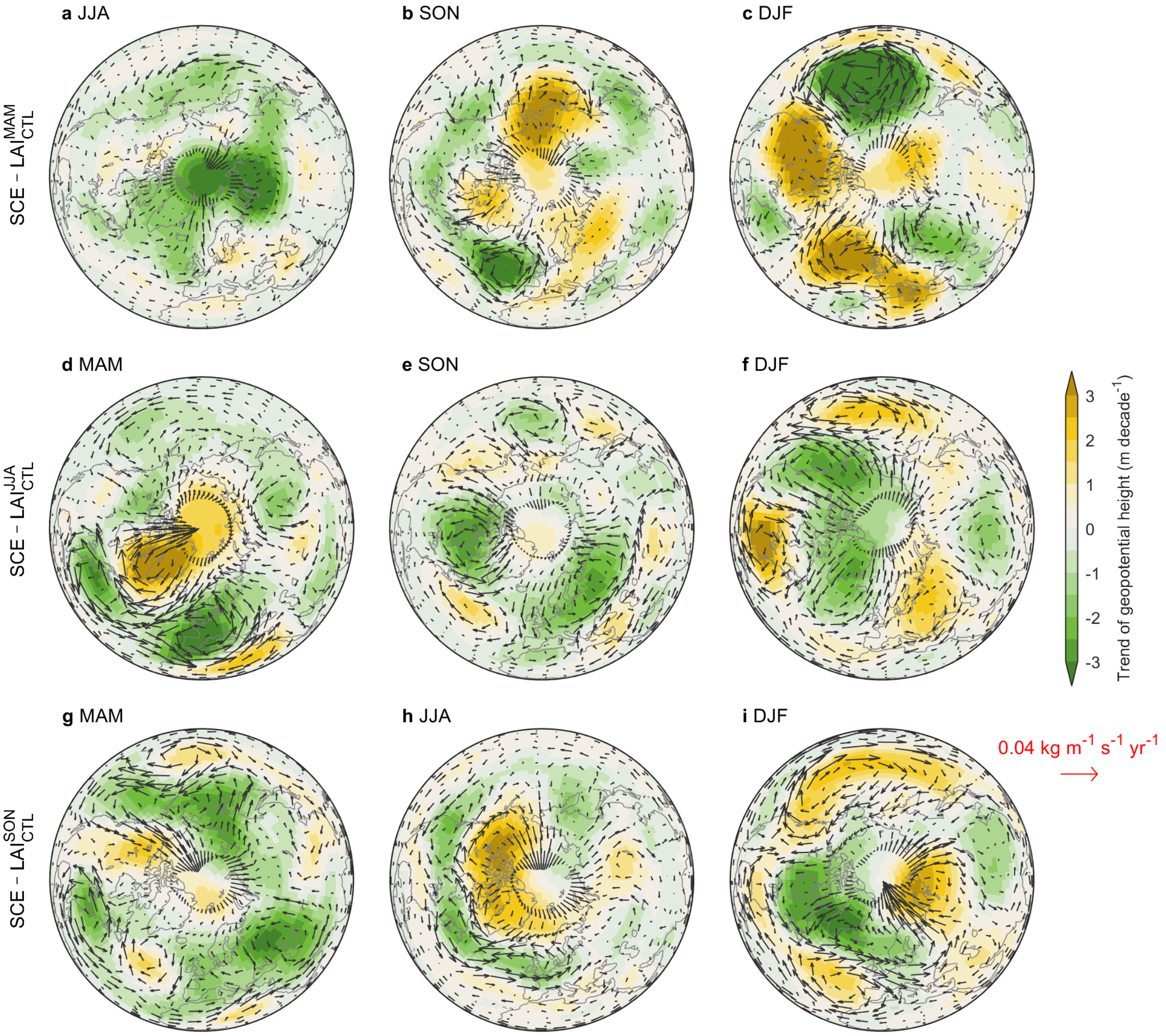

Supplementary Figure 13. Spatial patterns of the linear trends in 500 hPa (z500) geopotential height (shading) and wind speed (arrows) induced by MAM (**a-c**), JJA (**d-f**), and SON (**g-i**) LAI changes over 1982−2014. The changes were calculated as the difference between SCE and the simulation with fixed seasonal LAI (LAI^{MAM}_{CTL} , LAI^{JJA}_{CTL}, and LAI^{SON}_{CTL}).

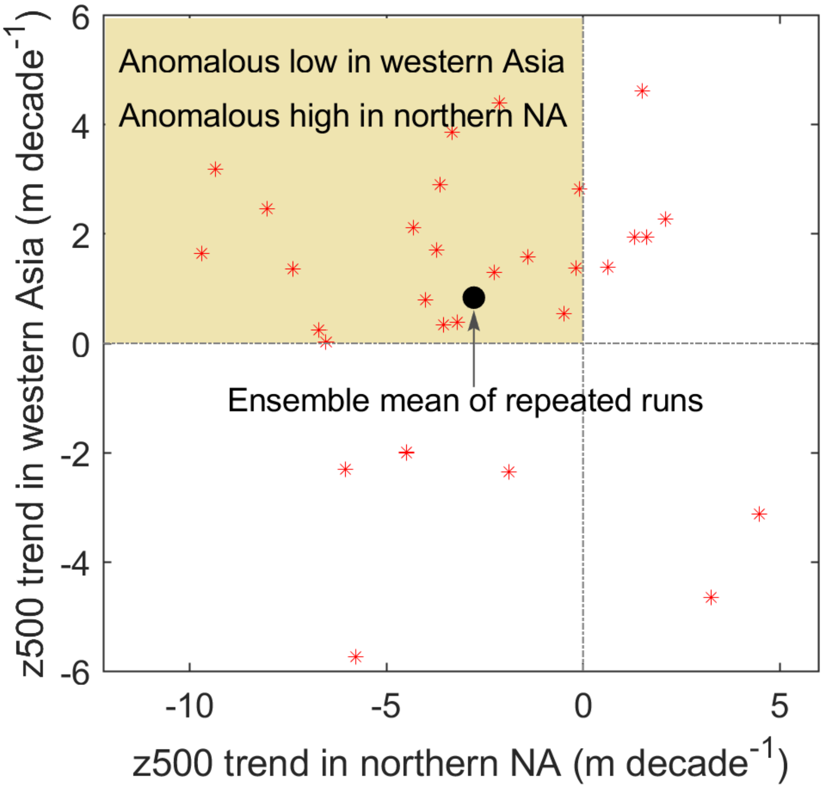

Supplementary Figure 14. Simulated trend of MAM 500 hPa (z500) geopotential height in western Asia (34°N−60°N, 34°E−75°E) versus that in northern North America (NA, 52°N−67°N, 97°W−161°W), driven by JJA LAI changes observed over 1982−2014. Red symbols represent the individual repeated runs (30 in total) forced by different initial conditions, and the black dot represent the mean value of the 30-member ensemble.
